# FACTORS AFFECTING SUICIDAL IDEATION IN COMMUNITY-DWELLING OLDER ADULTS WITH DEPRESSION

**DOI:** 10.1093/geroni/igad104.2148

**Published:** 2023-12-21

**Authors:** Shelby Smith, Lisa Barry, David Steffens, Richard Fortinsky

**Affiliations:** University of Connecticut, Farmington, Connecticut, United States; UConn Center on Aging, Farmington, Connecticut, United States; University of Connecticut School of Medicine, Farmington, Connecticut, United States; University of Connecticut, Farmington, Connecticut, United States

## Abstract

Suicidal ideation (SI) within the older adult population represents a continuing public health crisis. The previously published 5D Framework utilizes a set of five domains (depression, disability, disconnectedness, disease, and deadly means) to explain multifaceted influences on late-life suicide. This study explored factors associated with SI among community-dwelling older adults with depression by including measures of selected 5D Framework domains and additional factors potentially associated with SI in an explanatory analytic model. We utilized baseline data from an ongoing clinical trial to assemble a cohort of older adults (age 65+) who screened positive for depression via provider diagnosis or scoring ≥16 on the Center for Epidemiologic Studies Depression scale (CES-D). Individuals joining the clinical trial between May 2017-March 2020 completed baseline assessments evaluating levels of depression, cognitive impairment, psychological resilience, dispositional optimism, social engagement, level of financial strain, and educational attainment. Sl was defined as endorsement of Question 9 of the PHQ-9. In the study cohort (N=171), mean age was 73.5 [SD: 5.76], 67.3% female, 92.4% non-Hispanic White, and 18.1% endorsed SI. In a multivariate logistic regression analysis, increased severity of depression was positively associated with SI (AOR 1.18 [95% CI:1.06-1.31]) while higher level of optimism was inversely related with SI (AOR 0.913 [95% CI:0.84-0.96]). No statistical significance was noted for age, gender, education, financial strain, resilience, cognitive impairment, or social engagement. We conclude that optimism is a protective factor against SI, and reporting more severe depressive symptoms represent an important risk factor for SI, among older adults with depression.

